# Buffalo Academy of Medicine

**Published:** 1905-02

**Authors:** Franklin W. Barrows


### SOCIETY PROCEEDINGS.

### Buffalo Academy of Medicine.

*Section on Medicine, December 13, 1904.*

REPORTED BY FRANKLIN W. BARROWS, M. D., Secretary.

At the stated meeting of the Academy, December 13, the following program was presented by the Medical Section.

Dr. D. L. EDSALL, of Philadelphia, read a paper entitled,

### THE DIETETIC USE OF PREDIGESTED LEGUME FLOUR PARTICULARLY IN ATROPHIC INFANTS. WITH A STUDY OF ABSORPTION AND METABOLISM.

(AUTHOR'S ABSTRACT.)

The study was undertaken largely for the purpose of determining whether it is possible to give to infants that have difficulty in digesting a sufficient amount of milk proteid an amount of vegetable proteid that would be of nutritive importance to the child. A series of cases of atrophic infants were given bean flour in their milk mixtures, the flour having previously been predigested by means of a diastatic ferment. The clinical results, considering the class of patients, were in most instances remarkably good, the condition of the bowels usually improving and a large proportion of the babies making very rapid and good gains. The studies of absorption also show that the bean proteid in this form was quite as well absorbed as the milk proteid. A nitrogen metabolism experiment was also carried out on an adult. When bean flour was substituted for about an equal food value of milk the patient gained quite as well as he had on the previous diet and also showed somewhat more nitrogen retention than he had on the previous diet. The absorption in this instance was very slightly less good, this being perhaps due to the fact that there was some digestive disturbance at first when a large amount of bean flour was suddenly added to the diet. The reason for this favorable influence of bean flour perhaps does not lie solely in its large nitrogen content. It seems wholly possible that it is due to the nature of the proteid in the bean, the relation between this and the proteid of the nuclei of animal cells being sufficiently intimate to suggest this.

DISCUSSION.

Dr. Irving M. Snow.—The importance of the proteid content of food in its relation to metabolism, is shown by the fact that the human infant, which receives 1½ per cent. of proteid in its food, doubles its weight in three or four months, while the dog, with 4 per cent. of proteid in its food, doubles its weight in three or four weeks. I have never seen infants gain so rapidly, under any method of feeding, as in the cases that Dr. Edsall has reported tonight. I have often thought that the principles of infant-feeding might with advantage be applied to the feeding of adults in such conditions as are often found in typhoid fever and other diseases.

Dr. Dewitt H. Sherman.—In beginning to feed infants with this legume flour, what is a safe percentage to use? Is this food constipating, like barley gruel; and how does it compare in strength? How much of the diastatic ferment is added to the legumes? Was beef juice used in any of the cases reported? What are the dangers of scorbutus under this diet?

Dr. Allen A. Jones.—Dr. Edsall has added another most valuable research to his already excellent work in dietetics. Is the legume diet likely to cause great flatulence? Dr. Shattuck, of Boston, has, some time since, set us thinking on the importance of changing the diet from milk to some other food in cases of typhoid fever, where milk seems to disagree. In certain cases the tympanites, coated tongue and other unfavorable conditions disappear when another food is substituted for milk.

Dr. Charles S. Jones.—It often happens in the intestinal disorders of infants that a complete change of food improves the child, regardless of the virtues of that particular food. Does this fact account, in any degree, for the success of the legume diet? I knew of a serious case of infantile scorbutus cured on plantose and orange juice. Plantose, being a vegetable albumin, appears to be more easily assimilated and to cause less uric acid than the animal albumins. Is this true also of legume?

Dr. M. Hartwig.—Were any of Dr. Edsall's cases fed on predigested milk before his experiments with milk modified with the predigested legume? Did the doctor compare the results of his method of feeding with those obtained by feeding milk predigested by cereals or otherwise? In predigesting, were any other than starch digesting ferments used?

Dr. ARTHUR W. HURD.—Has this food been given for the relief of cases in which diarrhea was a principal feature? Was any other treatment given for the diarrhea while the food was being used?

Dr. EDSALL, in closing.—We generally start a baby on 2 per cent. of the bean flour, which is substituted for an equivalent amount of milk. The food controls diarrhea, probably like Nährzucker, through its dextrin content, this perhaps having a soothing action on the bowel,—like that of starch paste in proctitis. Beans have a very high percentage of nitrogen,—23 per cent. as compared to 8 per cent., contained in wheat. They contain much more proteid, then, than barley or any other cereals except other legumes. They rank fourth among all other foods in point of cheapness. White kidney beans were used in these feeding experiments. Forty grams of bean flour were predigested with five grams of a malt diastase. No other ferment was used in the food. Probably many of the available diastatic ferments would answer equally well. The food might cause scurvy without a proper admixture of the antiscorbutic foods. Most of the children in the series of cases reported tonight had, previous to these experiments, been fed on milk containing other cereals. No medication was used to control diarrhea. The children did not show flatulence while using this food. Indeed, the flatulence so frequently resulting from a leguminous diet seems due to the difficulty of digesting legumes, and predigestion seems to prevent this result. Plantose, referred to by Dr. Charles S. Jones, has a high percentage of nitrogen, but it is very insoluble. It is doubtful if it produces less uric acid than does meat. In typhoid fever, the difficulty with the assimilation of milk is usually due to bad milk. If, however, unfavorable intestinal symptoms occur during the use of perfectly good milk, I would modify the milk as I would for a child. This course has proven successful in many cases under my care.

Dr. FRANK W. LOVE read a paper, entitled,

### ETIOLOGY AND SYMPTOMATOLOGY OF CEREBRO-SPINAL MENINGITIS.

(AUTHOR'S ABSTRACT.)

*Synonyms.*—Cerebro-spinal fever, spotted fever.

*Etiology.*—The specific organism, diplococcus intracellularis meningitidis, is a possible variety or a degenerated form of the diplococcus of pneumonia,—Fränkel diplococcus. Infants and young adults are most subject to the disease, which, however, attacks all ages. It prevails most in the temperate zone and during spring and winter. Unhealthy and crowded habitations, mental and physical exhaustion, are predisposing factors. It is sporadic, endemic, or epidemic, as it was in the east and in New York city last winter.

*Symptomatology.*—It attacks the central nervous system and symptoms are referred to that. If brain cortex is involved it causes muscular tremor and paralysis. If the base is involved, pressure on cranial nerves affects special senses. Pressure on spinal cord causes hyperesthesia, and the like. Combined and mixed symptoms occur when both brain and cord are involved. The incubation time is unknown.

*Prodromal Symptoms.*—Patient is stricken suddenly, possible early lassitude, headache, vomiting, joint and myalgic pains.

The malady follows the rule of infectious diseases,—intense headache; backache (cervical and lumbar) are characteristic: chill; fever of moderate type; myalgia; joint pains and inflammation; convulsion; maniacal and excited tendencies; apathy; somnolence; stupor; coma; death. The fever in the fulminating variety, high,---105° F.; other forms are moderate,---101 to 103°.

The pulse is accelerated, slower at first, later 120-140. Early, of good volume, later, soft and compressible; in serious cases, small and feeble. Some clinicians believe that a pulse lacking tension is diagnostic. There is leucocytosis. The respiration is slightly increased; as the respiratory centers are involved, slow, sighing—Cheyne-Stokes—breathing; rapid breathing; possibly pneumonic complications.

The headache is intensified by light and noise; the patient groans even when comatose. There are upper cervical and lumbar spinal pains, myalgic pains in the abdomen and extremities, cephalalgia is associated with vomiting, hyperasthesia follows anesthesia, irritability, delirium, hallucination, apathy, stupor and coma occur; contracting muscles cause characteristic positions,—forearm on arm, leg on thigh, thigh on abdomen; back muscles cause orthotonus more constantly than opisthotonos. There may be photophobia,—variable pupil, strabismus (temporary), ptosis, possibly conjunctivitis, keratitis, purulent irido-choroiditis; tinnitus, aurium and otorrhea may occur; skin and visible mucous membranes in the invasion period show pallor and lividity; herpes facialis is, perhaps, diagnostic. Petechial eruption and pallor of the skin give the name spotted fever. The gastrointestinal symptoms include early vomiting, of nervous origin. Most types have anorexia, almost normal tongue, constipation, retracted belly. Typhoid type has diarrhea and tympanites. Rectal and vesical incontinence occur in the stage of muscular paralysis; arthritis occurs in some types.

"Kernig's sign" is slow and uncertain as to time of appearance. Constant watching for the sign is necessary. Its absence does not exclude diagnosis of cerebro-spinal meningitis.

*Types of the Disease.*—(*a*) Fulminant or malignant: sudden onset; very rapid coma state; omits earlier symptoms; lasts from seven hours to one or two days; is fatal; (*b*) typhoid type; typhoid state with meningitis and spinal symptoms; (*c*) intermittent variety: severe symptoms, remit or intermit, every day or second day; distressing symptoms subside and appear in twenty-four to forty-eight hours, increased in severity; (*d*) abortive form: severe and sudden onset, but immunity is established and disease checked after a day or two; (*e*) mild form: symptoms so mild and undeveloped that it is recognised only in an epidemic.

Dr. RAY H. JOHNSON read a paper entitled,

### REPORT OF A CASE OF MENINGITIS.

(AUTHOR'S ABSTRACT.)

F. W., boy, 17 months old, youngest of three children. Other two in best of health, were during dentition, and are exceptionally well-developed children.

The little patient, prior to the attack herein described, passed a perfectly normal childhood, with the exception of two troublesome teeth, which appeared about this time. For some time prior to April 29, 1904, the diet of the child had been condensed milk, Eagle brand. Upon this day the mother noted that the can she opened had a protruding top, as though fermentation had begun. Not knowing that it might harm the child, he was given the usual amount and put to bed in apparently good health. During the night the mother was awakened by the child crying and vomiting. A severe convulsion, lasting half an hour, followed. The convulsive movements were most pronounced on the right side. The following day, April 30, the child played around and seemed as well as usual until 2 p. m., when he had a second convulsion. Within the next seventy-two hours he had fifteen severe convulsions, which resulted in a left side hemiplegia,—affecting left side of face as well as left side of body. He was in a semi-conscious state, refusing all food except egg-water. The temperature was stationary at about 101° F. Bowels moved often (result of cathartics), the dejecta having a very offensive odor and containing much undigested milk curd. There was very little vomiting, a fact I had great cause to be thankful for in the weeks following.

About twenty-four hours after the last convulsion the temperature began rising until it reached 104° F., continuing between 104°

and 105°, except when influenced by hydropathic treatment, for a period of about three weeks. The pulse was 145, small and wiry. The bowels moved regularly. Passages were of a normal character and apparently not a factor in the continued high temperature. During this period there were continuous spasmodic actions of right arm and leg. The head was turned to the right side, and it was with great difficulty that the child took a sufficient amount of food. Paralysis of the left arm and left leg were complete, and the symptoms were so grave that a consultant suggested a diagnosis of tubercular meningitis. After three weeks of continued high temperature all symptoms slowly subsided. The temperature was normal in about four days, and in two weeks the child was able to sit up. Spasmodic movements of the right side subsided, the leg nearly recovered and the arm improved 50 per cent.

The paralysis of the left side and convulsive movements of the right side returned. This marked the beginning of another period of three weeks' suffering pitiful to witness. The temperature, instead of following the course of the previous attack, was normal, and at times subnormal. The basilar involvement was so great that it was impossible for anything to pass the throat, and no medicine, food or water entered the stomach for fourteen days. A sufficient amount of peptonised food was given per rectum to fairly well sustain the child. During these three weeks it was difficult to produce sleep, and during most of the waking hours he cried continually. Examination of the urine was made at frequent intervals and a trace of albumin was found at each examination; no casts were ever found; specific gravity and amount were in direct proportion to the amount of liquid diet and drinks taken. The convalescence from this attack, if it may be so called, was less rapid than the preceding one, and was without special interest.

A recent examination of the child showed marked improvement. He is now able to walk, although the control of the left leg is not perfect. He has that swing of the leg that is characteristic of such cases. About 25 per cent. improvement has taken place in the arm. The mental condition is as good as when the child was taken sick, and his vocabulary is gradually increasing, and is now larger than at the time his illness began. I have carefully looked for manifestations of brain irritability, but have found none.

DISCUSSION.

Dr. Allen A. Jones.—The last two papers have conveyed an excellent idea of this disease. Dr. Johnson's case, a most interesting one, acted very much like tubercular meningitis. If lumbar puncture had been made the diagnosis would most likely have corroborated this idea.

Dr. Dewitt H. Sherman.—I believe I accomplish less in the treatment of meningitis than in any other cases. Lately, I have used adrenalin solution for its action on the capillaries. Hypodermatically, adrenalin causes anemia of the part but has no great influence on the capillary circulation. To affect the capillaries it would seem necessary to introduce adrenalin intravenously. Normal saline hypodermoclysis seems to offer promise as a remedial measure in meningitis.

The Academy adjourned.

---

### Fakir, Pure and Simple.

JUSTICE SO CALLS "DOCTOR"---GIVES HIM THREE MONTHS.

In sentencing Vitorio Salamone to three months in the penitentiary, Justice McKean, in special sessions recently, said to the prisoner:

"Salamone, you are a fakir, pure and simple; you are a humbug, a fraud and a charlatan; a big, strong man like you should be at work and not trying to delude ignorant people. From the testimony in this case, one would think we were back in the fourteenth century."

Salamone was arrested on December 3, at No. 28 Bayard street, where, calling himself a grocer, he had a combination of a grocery store and a doctor's office. Here he charged five dollars for each consultation.

Lillie Enrico, of No. 180 Broadway, said that she called on Salamone on December 2, telling him that a sister-in-law had had a witch make her ill. Salamone said he would question the spirits, and after some peculiar actions gave the woman a prescription, which could not be filled at any drug store.

The complaint that Salamone was illegally practising medicine was made by the board of education to James Taylor Lewis, counsel for the New York State Medical Association, some weeks ago, and it was through the efforts of Mr. Lewis that the necessary evidence to convict was obtained.—*New York Tribune.*

---

TO PREVENT FALLING HAIR.

Walsh has used the following with gratifying success in preventing that condition so annoying to humanity which is described by the patient as: "my hair is falling out."

| | | |
|---|---|---|
| ℞ | Salicylic acid | 12.00 [ʒiii.] |
| | Carbolic acid | 4.00 [ʒi.] |
| | Castor oil | 12.00 [ʒiii.] |
| | Alcohol, to make | 180.00 [℥vi.] |

Mix. Directions—Apply freely to scalp once or twice a day.

